# Diet and Aging: The Role of Polyphenol-Rich Diets in Slow Down the Shortening of Telomeres: A Review

**DOI:** 10.3390/antiox12122086

**Published:** 2023-12-07

**Authors:** Stefania D’Angelo

**Affiliations:** Department of Medical, Movement and Wellbeing Sciences, Parthenope University, 80133 Naples, Italy; stefania.dangelo@uniparthenope.it; Tel.: +39-081-5474672

**Keywords:** Mediterranean diet, oxidative stress, antioxidant, telomere length, polyphenol, aging, nutrition, longevity

## Abstract

The ends of human chromosomes are defended by DNA–protein complexes named telomeres, which inhibit the chromosomes from fusing with each other and from being known as a double-strand break by DNA reparation proteins. Telomere length is a marker of biological aging, and disfunction of telomeres is related to age-related syndromes. Telomere attrition has been shown to be accelerated by oxidative stress and inflammation. Telomere length has been proven to be positively linked with nutritional status in human and animal scientific research as several nutrients influence it through mechanisms that imitate their function in cellular roles including oxidative stress and inflammation. Data reported in this article support the idea that following a low-in-fat and rich-plant polyphenols food diet seems to be able to slow down the shortening of telomeres.

## 1. Introduction

Aging is a gradual, time-dependent biological deterioration that results in an improved risk of cell death or epidemics [1]. Neurodegenerative disorders, chronic lower respiratory tract disease, heart disease, cancer, hypertension, and stroke are the leading causes of mortality among human aging-related deaths [2]. There are many physiological alterations that occur over time, such as epigenetic anomalies, genomic instability, loss of proteostasis, anomalous absorption of nutrients, altered intercellular communication, cellular senescence, and shortening of telomeres [3]. Telomeres are the physical terminals of chromosomes consisting of a non-coding DNA sequence that stabilizes the genome by making the end of the chromosome free from breaks and proximity [4]. With each cell division, telomeres shorten. The shorter the telomeres, the faster the cells age [5]. Telomerase, a ribonucleoprotein enzyme, has a DNA polymerase action and using a part of RNA as a template, can synthesize telomeric repeats at the ends of chromosomes, which prevent this chromosomal shortening. The enzyme telomerase is active in stem cells and could hypothetically function in all cells and, if its action were to increase, the shortening of chromosomes would be slowed down [4].

External factors, such as lifestyle, nutritional intake, genetic mutation, and heredity, can affect aging [6]. Studies carried out on twin subjects have shown that only 20–30% of lifespan is conditioned by genetics, while a large part depends on individual behavior and environmental factors. Some age-related diseases such as obesity, insulin resistance, cardiovascular syndromes are associated with an increase in inflammation and oxidative stress, and a decrease in telomerase activity and telomere length (TeLe) [7]. Some lifestyle habits, such as smoking, poor physical activity, psychological stress, exposure to pollution, and unhealthy diets, can significantly raise oxidative stress and indirectly accelerate aging, as the shortening of telomeres is also caused by oxidative stress [5].

The type of diet can affect the activity of telomerase and therefore cause a shortening of the length of the telomeres. 

Polyphenols are phytochemicals present in vegetables and fruits and represent a large set with more than 8000 identified molecules. There is a convincing sign to suggest that these phytochemicals can improve cellular antioxidant protection and therefore increase the defensive impact against age-related disorders [8]. Numerous molecules of the polyphenolic group act as strong antioxidants in vivo and in vitro and prevent damage from oxidative stress. By influencing different signaling pathways, they can support their anti-aging activity by extending lifespan. Polyphenols have been revealed to exert positive outcomes on many age-related syndromes, which also influence TeLe. It has also been confirmed that the relationship between polyphenolic molecules and apoptosis is the main goal for raising life expectancy [9].

The studies reviewed in this paper support the idea that following a low-fat, polyphenol-rich diet, as well as engaging in repeated physical activity and decreasing stress, appears to be able to slow the shortening of telomeres.

## 2. Methodology

The most important life-science databases of references and abstracts (PubMed, MEDLINE, Web of Science platform, and Google scholar) were methodically analyzed (from 2013 to October 2023), using the terms “polyphenols”, “phytochemicals”, “oxidative stress”, “nutrition”, “antioxidant”, “Mediterranean diet”, “diet”, “longevity”, “aging”, and “telomere length”, and refining the results for “human studies” AND “controlled trials”. Boolean operators “AND” and “OR” were used alone or as combined descriptors, by removing duplicates and non-relevant papers.

## 3. Telomere Length: A Marker as a Biological Clock

The constant increase in life expectancy has totally changed people’s way of thinking; in fact, populations would like to live longer but also healthier. Therefore, researchers are investigating solutions for healthy aging, understood as physical and cognitive functions preserved in the individual, in the absence of major chronic diseases, intact mental health, and therefore, ultimately, a good quality of life which therefore appears to be one of the main public health concerns. It is also important to underline how the costs for a progressively aging population represent an enormous and constantly increasing health burden [10]. Trying to prevent, or slow down, the onset and progression of age-related diseases is currently of fundamental importance in public health [11]. Although the biological causes of human senescence remain largely unknown, in recent decades studies have proven the existence of common cellular and molecular traits associated with aging [12,13].

Therefore, currently, finding markers to better characterize the aging process has become a priority. López-Otín and colleagues [12] have shown nine main signs characterizing aging: epigenetic anomalies, genomic instability, unregulated absorption of nutrients, loss of proteostasis, alteration of mitochondrial function, abnormal intercellular communication, cell senescence, depletion of stem cells, and attrition of telomeres [11].

Telomeres are repetitive DNA sequences (5′-TTAGGG-3′) found at the ends of eukaryotic chromosomes that allow cells to differentiate chromosome ends from double-strand breaks and thus defend chromosomes from end-to-end fusion, recombination, and degradation [11].

The double helix of DNA, when it unwinds and separates so that the information contained in it is copied, never replicates completely, and with each duplication cycle, the genetic material shortens, losing the terminal part. It would understandably be very serious if telomeres, sequences of bases that do not have any information, did not exist, so as not to cause damage even if progressively shortened. 

Telomeres are stretches of DNA that protect strand integrity and can be thought of as a specialized cap of chromosomal DNA. During various cell divisions, telomeres avoid the loss of DNA base pairs. Over time, however, the length of the telomeres decreases until this cap becomes too short and the cell is no longer able to divide, causing senescence. This phenomenon is a replication problem [14,15].

Due to the problematic of conclusive replication, telomeres shrink in each generation of the cell until they reach a substantial length in the crisis phase of aging [16]. At this stage, cell division significantly slows down, resulting in slow cell death. This phenomenon is named “replicative mortality”. Cells involved in growth and reproduction (stem cells, eggs, and sperm) synthesize considerable amounts of the enzyme telomerase, an enzyme concerned in preserving the length of telomeric DNA [17]. Telomerase, an enzyme with a catalytic unit called protein reverse transcriptase, therefore serves to combat the problem of final replication through the lengthening of telomeres [15,18]. Most adult cells express little or nothing of this enzyme, causing them to age and eventually die [19].

At each cell division, the terminal ends of the chromosomes are subjected to friction, which is the cause of their progressive shortening [20], and therefore, the length of the telomeres is considered a marker of aging [21].

Showing hallmarks of aging has helped conceptualize aging research and hinted at the tantalizing prospect of delaying multiple age-related syndromes by targeting the aging process [22]. Although TeLe, and its attrition over time, is very inconstant between people, it is thought to be stable from childhood through young adulthood but begins to decline in older adulthood [14]. Shorter telomeres are linked to reduced life expectancy and increased rates of development of age-related syndromes [21]. Telomere shortening and damage are documented causes of cellular senescence and aging. Furthermore, leukocyte TeLe is positively linked with the number of years of healthy life. Numerous human conditions associated with standard aging are precipitated by faster telomere dysfunction [15,23].

The friction suffered by telomeres is accelerated by phenomena such as oxidative stress and inflammation [3,24]. Levels of mitochondrial alteration, oxidative stress, antioxidants, inflammation, telomere shortening, and genetic mutations all play a fundamental role in defining cellular aging. Studies suggest that the free radical’s synthesis and oxidative stress by it play a crucial role in shortening telomeres by decreasing the activity of telomerase or telomere repeat binding factor 2 (TRF-2) levels [9]. Telomeres shorten more rapidly under conditions of oxidative stress and inflammation. Free radicals are in fact capable of directly attacking the terminal parts of DNA, damaging them and indirectly damaging telomerase, with a synergistic shortening and aging effect. And so, correlations are seen between chronic diseases and shorter telomeres, from Parkinson’s to type I and II diabetes.

But scientific research has suggested how telomere attrition can be changed [11,25] and the variability in TeLe can be partly conditioned by lifestyle practices, including dietary patterns.

Since plant-based foods are known to have antioxidant properties, and TeLe is affected by both processes, researchers believe there are reasons why consuming plant-based foods may help slow the attrition that occurs checks on telomeres over the years.

For this reason, in this narrative review, the most recent evidence on the link between consumption of edible plants and TeLe has been summarized. Data reported in observational studies on the link between TeLe and adherence to plant-rich dietary patterns principally focused on the Mediterranean diet (MedDiet) [11].

Changes in diet and lifestyle can change the activity of telomerase in peripheral blood mononuclear cells [26], although it is not clear whether this phenomenon can intervene indirectly in changing TeLe [27].

Certainly, healthful lifestyles and diets are beneficially linked with TeLe. Of interest to nutritionists, TeLe has been revealed to be linked with nutritional status in animal and human studies. 

If inflammation and oxidative stress shorten, there are molecules capable of increasing the stability of telomeres and slowing down their degradation, such as polyphenols. Plant chemical compounds, such as resveratrol, curcumin, quercetin, and catechins, which are richer in some plants (e.g., apples, pears, cherries, berries, peppers, carrots, tea, coffee, cold-pressed olive oil, garlic, onions), act as powerful antioxidants offering greater stability to telomerase and greater TeLe. So, if it is true that as you age, everything gets shorter, it is also true that the process can be slowed down with an elixir of long life that is not so difficult to find: philosophical cups of fruit and vegetables.

## 4. Nutrition, Bioactive Phytochemicals, and Longevity

Numerous scientific studies have tried to define the main creators of human longevity. Longevity depends, in different proportions, on both environmental and genetic factors [28]. Although it is complex and difficult to define with certainty, several studies seem to assert that precise dietary patterns are possibly linked to longevity [29]. In recent years, the nutritional habits of five populations living in areas of the Earth labeled as “Blue Zones” have aroused interest, as they are characterized by widespread and extraordinary longevity. The “Blue Zones” are Sardinia, Italy; Okinawa, Japan; Loma Linda, California; Ikaria, Greece; and the Nicoya Peninsula, Costa Rica; and these are areas of our planet that are inhabited by populations with a high prevalence of centenarians and who preferentially follow plant-based diets [8,30]. Although the data reported in the current scientific literature on diet and lifespan are complex and often divergent, there are competent studies such as the Seven Countries Study on Mediterranean diet and the Dietary Approaches to Stop Hypertension (DASH) [31] that have estimated the effect of diet on the development and progression of some diseases in an extremely convincing manner.

Regardless of different scientific investigation methods, the data presented support the idea that diets rich in vegetables and fruit, and thus with a high intake of phytonutrients with antioxidant and anti-inflammatory action, and a reduced intake of meat, refined cereals, saturated fats, sugar, salt, and fatty dairy products, are linked to a low incidence of genetically non-transmissible diseases [8,32]. In recent years, scientific data relating to a possible connection between nutrients or bioactive foods, whole foods, or specific dietary models and TeLe have increased considerably. Most studies have strengthened the concept that nutritional protocols that include a high intake of plant foods are an interesting tool capable of slowing down the shortening of telomeres.

In 2011, one of the first reviews was published that tried to understand a possible connection between nutrition, diet, and TeLe [27]. One of the authors’ main assertions was that many plant-based substances, such as folate, polyphenols, vitamins E and C or curcumin, might be able to slow down the friction of telomeres. In the following years, this topic was increasingly addressed by a greater number of researchers.

Freitas-Simoes et al., focusing on nutrition (foods, nutrients, and dietary patterns) and TeLe, concluded that antioxidants ingested through food and the consumption of foods rich in phytochemicals help to slow down the shortening of telomeres. Instead, the intake of saturated fats, refined cereal flours, and meat and derivative products and the use of sugary drinks seemed to handle the presence of shorter telomeres [33].

Data reported in a systematic review published in 2017 support the health benefits of adhering to the MedDiet on TeLe. Data relating to fruit and vegetables confirmed a good association between intake of these foods and TeLe. Some food categories, counting processed meats, grains, and sugary drinks, were found to be associated with shorter TeLe. The researchers, however, underlined the need for research that would take into consideration further epidemiological data and clinical studies, to develop further confirmations in this regard [34]. 

Data reported in a 2018 review strengthened the link between sugary drink consumption and telomere shortening, and a new association between TeLe and coffee consumption was discovered. Controversy persisted over meat eating and TeLe, largely because of the supposedly diverse results of whole meat and processed meat. In overall terms, the authors asserted that the accumulative consumption of antioxidant-rich plant foods was related to the maintenance of TeLe and that, in any case, dietary intervention studies with results on TeLe were still few [33]. In the prospective cohort study “Nurses’ Health Study”, involving 121,700 nurses, greater adherence to the Mediterranean diet related to longer telomeres in leukocytes. These findings further supported the benefits of adhering to a plant-based diet to promote health and longevity [11].

In a recent review, Crous-Bou et al. have summarized the study data relating to the action of dietary patterns rich in plant foods on TeLe. Although the evidence extrapolated from clinical studies is very limited, the authors concluded that many observational studies supported the beneficial action of adhering to a MedDiet (a plant-rich dietary pattern), consumption of seeds (and its derivatives) and carotenoid-rich food intake on TeLe. These results confirmed the beneficial action of a diet rich in plants and plant foods on longevity and health [11].

Nutrients and bioactive molecules ingested through edible plant foods act synergistically to influence different processes (e.g., inflammation, telomerase activity, oxidative stress, and DNA methylation), all physiological processes related to telomere attrition [27].

Since lifestyle and diet can affect the individual’s inflammatory state and oxidative stress, phenomena involved and responsible for telomere friction, could also affect TeLe. Therefore, a healthy lifestyle and a diet rich in fruit and vegetables, combined with constant physical activity and non-smoking, may be able to slow down the shortening of telomeres [15,35].

In a small pilot study, 30 men with prostate cancer were asked to make comprehensive lifestyle changes, including a change in diet, preferring unrefined, low-fat plant foods supplemented with omega-3 fatty acids (from oil fish), soy, and vitamins E and C. After three months, researchers proved an increase in the activity of the telomerase enzyme present in peripheral blood mononuclear cells [26]. It was proven that the intake of dietary fiber, particularly from whole grains and cereals, was positively linked with TeLe [36]. In a multi-ethnic study on atherosclerosis, the consumption of processed meat showed, as was expected, an inverse association with TeLe [37].

### 4.1. Plant-Based Diet and Telomere Length

In recent years, there has been a clear increase in the amount of scientific research on the topic of diet and TeLe. This reiterates the growing interest in the scientific world in researching the anti-aging abilities of nutrition. Since a close link has been proven between oxidative stress and the friction responsible for the shortening of telomeres, it is plausible that the consumption of foods rich in antioxidants, and therefore a diet rich in edible plants, can counteract the friction of telomeres and so have important health benefits.

Lee et al. [38] studied dietary information on middle-aged and older Korean adults and showed two major nutritional patterns (using factor analysis). The authors observed that subjects who followed a prudent alimentary pattern (a high intake of whole grains, seafood, vegetables, legumes) in the distant past (i.e., 10 years earlier) showed a longer TeLe. However, no significant relations were present in subjects with a high adherence to the Western dietary model (characterized by an elevated consumption of refined cereals, red or processed meat, and sugary carbonated drinks) [38].

Gong et al. [39], after defining four main dietary patterns, reported that Chinese women who adhere to a “vegetable-rich” diet, characterized by a greater consumption of fruit, whole grains, various vegetables, nuts, eggs, and tea, showed significantly longer telomeres compared to women who followed the “macho” dietary pattern (consisting mainly of animal foods and alcohol), or the “traditional” pattern (i.e., red meat, rice, and pickled vegetables), or a “high energy density” (i.e., with sugary drinks, fried foods, and wheat flour) [39].

Karimy et al., in a cross-sectional study conducted on 300 individuals, residents in Tehran, proved that adhering to a healthy diet, characterized by a prevalence of fruit, integrated cereals, vegetables, and fish was essential to prevent the shortening of telomeres and therefore increase life expectancy. According to this study, adherence to a healthful diet was significantly linked with an increase in TeLe in all subjects; on the contrary, the Western dietary model related to a reduction in TeLe [40].

### 4.2. Telomere Length and Mediterranean Diet

In the last decade, much scientific research has tried to explain the molecular mechanisms underlying the positive effects on human health of the intake of micronutrients and macronutrients present in the various foods included in the Mediterranean diet [41,42,43,44].

The Mediterranean diet (MedDiet) is a dietary protocol proposed by the American doctor Ancel Keys in the 1960s and is one of the most studied dietary models in the world [45]. It is the typical dietary model of the inhabitants living in the Mediterranean lands. Historically, in the countries bordering the Mediterranean Sea, the diet included the intake of high quantities of vegetables, legumes, nuts, seeds, marginally refined whole grains and olive oil as a condiment [29,43]. Since the 1960s, MedDiet has been widely studied to understand its role in the prevention of many chronic and/or degenerative diseases [29].

The MedDiet is a dietary model that seems to have a fundamental role in the prevention of many chronic and/or degenerative syndromes. Considerable evidence supports its action in reducing the incidence and mortality in cases of cardiovascular disease, supports its action in reducing the risk of coronary heart disease and incidence of myocardial infarction and stroke, improves metabolic syndrome (significant inverse associations for blood pressure blood, waist circumference, and low levels of HDL-C) [46], improves its ability to act on diabetic patients (lower risk of type 2 diabetes mellitus) [47], and improves its ability to act on mental health (lower incidence of depression) [48]. Martín-Peláez et al., in epidemiological research, observed an association between MedDiet and the decrease in the incidence of cardiovascular syndromes [49]. Other observational and epidemiological studies have proven that there is an inverse relationship between the MedDiet and the risk of incidence of various types of tumors [50]. The famous interventional study PREDIMED (Prevención con Dieta Mediterránea) compared MedDiet with a traditional control diet and confirmed a reduced in the incidence of cardiovascular diseases and diabetes in MedDiet subjects [51,52,53].

Furthermore, greater adherence to this diet has also been correlated with a greater length of leukocyte telomeres, greater action of the telomerase enzyme, a reduced plasma level of proinflammatory cytokines (such as tumor necrosis factor alpha TNF-α and interleukin 6 and) and a reduction in oxidative stress [28,54].

Vasto et al., comparing the lifestyle of the inhabitants of two Mediterranean populations, in particular individuals who lived on the island of Ikaria (Greece) and others who lived in the Sicani Mountains (Sicily, Italy), proved a high longevity found in two populations probably related to a high adherence to the MedDiet [22].

A recent meta-analysis created in clinical and observational studies has proven the health actions of MedDiet on many chronic syndromes, such as myocardial infarction, cardiovascular disease, coronary heart disease, diabetes, cancer incidence, neurodegenerative diseases, and general mortality [55]. Furthermore, other studies have proven the properties of MedDiet on cognitive function, aging parameters, and improvement in general quality of life [56]. MedDiet is a nutritional protocol that has been linked to the concept of healthy aging, defined as aging with the absence of serious chronic syndrome, absence of depression, absence of pain limiting function, good mental and physical condition, good social functioning, and independent functioning of daily activities [57]. In general, it is possible to assert that individuals who follow the MedDiet have greater longevity [54]. Another study confirmed that greater adherence to this dietary protocol in older adults in the United States and Israel is linked to better physical and cognitive function; in these individuals, in fact, a better walking speed and less disability were shown [28,58].

Furthermore, in recent years, the MedDiet is considered an environmentally sustainable dietary pattern too [59], a very important property considering the importance that the concept of sustainability now has at the center of every social and economic policy.

The MedDiet stands for the set of foods generally consumed by populations living in the Mediterranean regions, with olive oil playing a fundamental role in the food pyramid, as it is recommended as the main source of fat due to its high nutritional qualities (extra virgin olive oil) [60].

The main characteristic of this diet is represented by a high intake of foods of plant origin and the consequent assimilation of a high concentration of phytochemicals. In fact, the traditional MedDiet is characterized by a high intake of fruit, nuts, vegetables, walnuts, legumes (as a source of vegetable proteins), and cereals (mainly unrefined, a source of complex carbohydrates); a high intake of olive oil, as already mentioned, as a source of lipids with a high percentage of unsaturated fatty acids; a recommended moderate consumption of fish; a reduced intake of dairy products, meat, and poultry; and regular but moderate alcohol intake (particularly red wine with meals).

The MedDiet as an example of a healthy diet and lifestyle has aroused great interest across the globe. In fact, currently, an ever-increasing number of studies are based on clinical and epidemiological investigations on the health effects of eating Mediterranean foods, studies which in some cases also involve populations living in territories outside the Mediterranean regions. However, it is important to underline how the beneficial effect of the Mediterranean diet on health may differ between Mediterranean and non-Mediterranean countries [61], perhaps because the usual concept of transferability concerns only food feeding and less so other aspects, such as ingesting food during the day, and food and culinary conjugation [53].

Considering the many studies that prove the beneficial effects of MedDiet on longevity, many scholars have agreed that the many nutraceuticals included in MedDiet are also responsible for this effect, partly thanks to the antioxidant and anti-inflammatory properties phytochemicals possess. Therefore, since vegetables, fruit, nuts, and olive oil (key components of the MedDiet) have well-known anti-inflammatory and antioxidant effects, and that TeLe can be affected by both processes, it was assumed that greater adherence to MedDiet, and consequent longevity, may be associated with a slowdown in telomere shortening. As proof of this theory, research has shown that leukocyte TeLe is longer in subjects taking MedDiet [62].

The first study that tried to prove this theory involved 217 Italian elderly people (average age: 78 years) [54], whose adherence to the MedDiet was evaluated using the MedDiet Score according to the method developed by Trichopoulou et al. [63]. Subjects with greater adherence to the MedDiet (score ≥ 6) showed statistically significant longer telomeres associated with those with lower adherence to this nutritional protocol [54]. Similar results were shown in a much larger study, conducted on 4676 women aged between 42 and 70 years, from the Nurses’ Health Study in the United States [11]: superior adherence to the MedDiet was linked with higher telomeres longer. The authors compared the combination between different dietary patterns and TeLe: a higher alternative healthful eating index (i.e., beneficial eating) showed a weak optimistic association with a longer TeLe, but they found no statistically substantial associations for prudent or Western eating patterns [11]. In cross-sectional research on 1743 subjects from a multi-ethnic American cohort (average age 78 years) [64], it was verified that better adherence to the Mediterranean nutritional protocol was linked with a longer TeLe in whites, but not in African Americans and Hispanics. More recently, cross-sectional research conducted on 520 subjects in the PREvención con DIeta MEDiterranea (PREDIMED) study (average age 67 years), described that greater adherence to MedDiet was linked with longer TeLe in women, while the opposite was shown in men [65]. Finally, no association was found between TeLe and adherence to the MedDiet, nor diet quality scores, in 679 older Australians (average age 63 years) [66,67].

Some studies have shown a positive association between adherence to the MedDiet and the length of leukocyte telomeres in two different population subgroups [11,53]. In detail, the populations considered were a group of elderly people from Southern Italy [54] and a group of nurses between 30 and 55 years old [54]. Likewise, comparing elderly Dutch and Greek men, the study reported that Greek subjects had longer leukocyte TeLe [68]. In studies in which telomerase activity was also measured in leukocytes, a positive association was proven between increased activity of this enzyme and TeLe [53]. As already written, oxidative stress and inflammation can also influence the speed of telomere shortening [68]. In this sense, it was proven how telomerase action was negatively modulated both by oxidative stress and by inflammation and oxidative stress [54]. In contrast, in a study comparing elderly Dutch and Greek men, no relationship was found between leukocyte TeLe and indicators of oxidative stress and plasma antioxidants, even if the endogenous serum antioxidants albumin and uric acid were positively related to the TeLe [68].

Another investigation involved elderly subjects who consumed three different diets for 4 weeks: one group followed a diet with saturated fatty acids, another group followed a diet low in fat and high in carbohydrates, and the third group of subjects followed a MedDiet enriched in MUFA (randomized crossover design). Human umbilical endothelial cells were incubated with each patient’s serum. Among the various subjects, a lower percentage of cells with telomere shortening was verified in the individuals who followed the MUFA-rich diet, compared to what was shown in the subjects who followed the other two nutritional regimes. Furthermore, this result was associated with a lower intracellular concentration of reactive oxygen species (ROS) and reduced cell apoptosis [69].

In 2020, Canudas et al. published the first meta-analysis showing a beneficial association between MedDiet and TeLe adherence in blood cells. The authors conclude by saying that, considering how the shortening of telomeres is also partly involved in biological aging, greater adherence to a healthy nutritional model such as the MedDiet can be an excellent preventive therapy to counteract multiple age-related syndromes, slowing down the shortening of telomeres. They state, of course, for completeness, that several more prospective observational studies and clinical trials, considering large populations, are needed to confirm the beneficial effect of MedDiet on both telomerase activity and telomere shortening [70].

In conclusion, however, it is possible to argue that adherence to the MedDiet, assessed through precise scores, is linked with a longer TeLe in many populations, even with diverse backgrounds. The modifications noted between women and men are particularly intriguing and will certainly need to be the subject of further research. Adherence to so-called “vegetable-rich”, “healthy”, or “prudent” dietary patterns (obtained by factor analysis of food and nutrient intake data) is linked to longer TeLe, an outcome that has not been shown for Western countries, whose populations predominantly follow a Western-style diet [67].

The molecular mechanisms that explain the multiple benefits proven in subjects who have a high adherence to a diet rich in vegetables have not been completely clarified. Nevertheless, numerous molecular and clinical pathways have been considered, suggesting possible helpful molecular changes induced by the Mediterranean dietary pattern. Some of the proposed mechanisms are common to diverse pathological conditions, such as the reduction in both oxidative stress and the chronic inflammatory state of subjects, mainly attributable to the effects of bioactive antioxidant components consumed through the staple foods of plant-rich diets.

The analysis of the oxidative state of the ninety-year-old and centenarian people of Cilento presented a slight intensification in reactive oxygen species (d-ROM test) (324.1) and results within the limits for anti-ROM 1 and 2 (234.4 and 1188.8), reflecting good overall control of the oxidative equilibrium [71]. These outcomes propose that genetics, the Mediterranean lifestyle (nutrition and psychosocial habits), could be decisive in supporting this unaltered balance over time, ensuring healthier aging [72].

## 5. Bioactive Phytochemicals Can Slow Down the Shortening of Telomeres: The Anti-Aging Action of Polyphenols

With the growing awareness of the impact that an adequate diet can have on health, more and more attention is being paid to the benefits for human health of the use of natural products, and in particular foods rich in phytochemical substances such as polyphenols. Polyphenols are the largest group of secondary metabolites without energy value and are synthetized by plants as a response to a stress situation [73]. Polyphenolic compounds have been defined as “essential for lifespan” precisely because of their recognized significant impact on health [74]. There are approximately 8000 polyphenols which have been divided into various classes based on their chemical structure. These classes, which include non-flavonoids or flavonoids, were further classified based on the number of hydroxyls and the type and position of other substituents, into flavonols, flavanols, anthocyanins, flavones, procyanidins, stilbenes phenolic acids, and tannins based on the number of hydroxyls in the molecule and the type and position of the other substituents. However, despite a different classification, there are main common structural characteristics which are represented by an aromatic ring and at least one hydroxyl group [75,76].

Food polyphenols are mostly present in plant foods such as fruit, vegetables, dried legumes, olives, cereals, tea, cocoa, and wine [77]. Plant sources also include bark, leaves, fruits, spices, berries, greens, roots, herbs, nuts and seeds, and whole-grain products, extra virgin olive oil, green tea, red wine, coffee, and turmeric. Some common food polyphenols are, for example, lignin from whole grains and nuts; pro-anthocyanidins present in grapes, pine bark and cocoa; anthocyanins/anthocyanidins in brightly colored vegetables and fruits such as berries; typical soy isoflavones; tannins in tea and nuts; catechins present in grapes, wine, and green tea; quercetin present in onions and grapes; and naringenin/hesperidin in citrus fruits and resveratrol in wines [78].

Wild and local edible plants, taken as raw or cooked salads, recommended in the MedDiet, have a high concentration of polyphenols, and are characterized by a high antioxidant power. For example, blueberries and red radicchio are among the vegetables in the MedDiet with the highest antioxidant power. Epidemiological data show how, for example, cardiovascular diseases have a low incidence among populations living in the Mediterranean area, a geographical area where plant foods rich in antioxidants are a significant part of the diet followed by the population living there [8].

**Figure 1:**
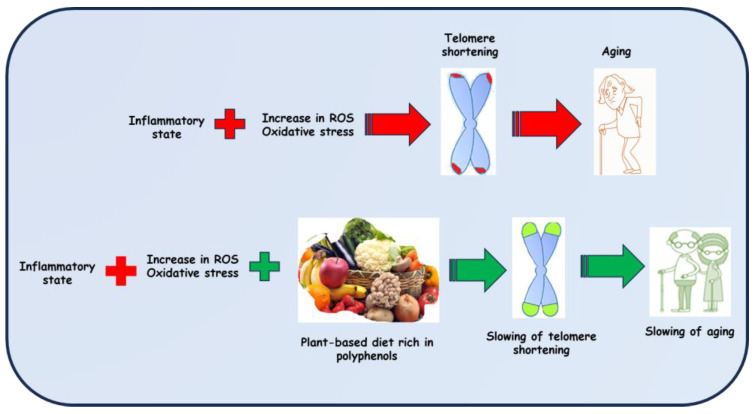
Polyphenols play an anti-aging role by preventing the shortening of telomeres with their anti-inflammatory and antioxidant properties.

Extracts obtained from edible plants can act on cellular damage associated with pathologies, on the generation of ROS and on physiological processes, such as metabolism and inflammation. Consequently, considering all these considerations, the radical trapping properties owned by the Mediterranean flora, characterized precisely by plant species endowed with anti-inflammatory, anti-aging, and antioxidant properties, deserve further investigation, and justify the search by scientists for new sources of natural molecules, capable of slowing down the shortening of telomeres (Figure 1).

Since it has been shown that TeLe is also influenced by an inflammatory/oxidative state, it follows that a greater intake of foods rich in antioxidants and/or greater adherence to an anti-inflammatory diet can play a role in slowing down the shortening of the length of the telomeres and therefore, in general, influence the health and longevity of individuals.

Research into the helpful health properties of polyphenols has increased significantly over the past two decades [79,80,81,82,83]. Polyphenols have shown anti-inflammatory, antiadipogenic, antimicrobial, antidiabetic, antioxidant, neuroprotective, and anticarcinogenic effects [84,85,86,87,88,89,90,91,92,93,94,95,96].

It has been shown that these phytochemicals can also regulate the composition of the intestinal microbiota [97]. Several studies, both in vivo and in vitro, have revealed that polyphenols have an ability to interfere with cellular signals, and to stimulate a bio regenerating response: this allows the stimulation of the synthesis of endogenous antioxidants [9,98].

Polyphenols have been shown to modulate the redox state of cells, can alter cell signaling, and help prevent the accumulation of damage in molecules such as lipids, proteins, and nucleic acids with a moderately long biological lifespan. This occurs both directly, through scavenging of ROS, and secondarily, through interaction with transcription factors that coordinate the antioxidant response. In fact, it has been shown that polyphenols stimulate the overexpression of antioxidant enzymes such as catalase and superoxide dismutase [99,100]. Polyphenolic molecules such as quercetin, resveratrol, and curcumin have a defensive role against damage from oxidative stress [8,9,101]. Therefore, through their anti-inflammatory and antioxidant abilities, polyphenols could also have a beneficial impact in slowing down the shortening of telomeres and helping the action of the telomerase enzyme. Nowadays, researchers are trying to increase telomerase action, stabilize TeLe, and extend life by using antioxidant supplements such as polyphenols [102]. And in fact, many studies suggest that polyphenols can influence the length of telomeres and prevent, as far as possible, their shortening. And this is further evidence of how diet is an important factor in deciding the state of TeLe.

Procyanidins and proanthocyanidins, oligomeric compounds formed from catechin and epicatechin molecules, exist ubiquitously in many edible plant sources including barley, hops, maize, grapes, apples, strawberries, almonds, cocoa, cinnamon, peanuts, and tea. Grape seeds supply a various source of proanthocyanidins, owing to the abundance of galloylated oligomers. These compounds are defined as powerful free radical scavengers; furthermore, in human lymphoblastic cells, their anti-inflammatory capabilities reduce apoptosis and prevent chromosomal damage induced by hydrogen peroxide. Similarly, dietary administration of grape seed polyphenols together with curcumin in mice induced the presence of longer telomeres [103]. The ability of these polyphenols to quench free radicals is 20 times greater than that found in vitamin E and 50 times more effective than vitamin C [104].

In male Wistar rats, the properties of modest consumption of red wine (equivalent to approximately 0.15 mg% resveratrol) or high (H, 400 mg%) or low (L, 0.15 mg%) resveratrol doses on slowing vascular aging and extending lifespan has been studied. The researchers proved an increase in TeLe > 6.5 times compared to the control, and an increase in telomerase activity even with the intake of low levels of resveratrol [105].

Several factors, such as telomerase and Werner syndrome helicase (WRN), contribute to the maintenance of telomeres. Current data show that resveratrol has the potential capability to slow telomere dysfunction by activating WRN helicase and telomerase, without disturbing cell proliferation [106]. Moreover, resveratrol delays senescence of endothelial progenitor cells, associated with telomerase activation and protein kinase B phosphorylation [107]. Lately, it has been proven that this polyphenol can influence the anti-aging method through the activation of telomerase via a nicotinamide phosphoribosyltransferase (NAMPT) and sirtuin 4 (SIRT4)-dependent pathway in human aortic smooth muscle cells. Consequently, the NAMPT-SIRT4 and human telomerase reverse transcriptase (hTERT) axis may be an innovative mechanism underlying the helpful abilities of resveratrol on cardiovascular disease [108]. Currently, there are no data relating to clinical studies proving the action of resveratrol on the maintenance of telomeres. However, there is research showing the possible effects of resveratrol on the maintenance of telomeres, which could also be relevant to improve human health and make this polyphenol an excellent candidate for anti-aging action [109]. It has been revealed that modulation of Sirt1 by resveratrol can influence mechanisms related to longevity [109,110,111]. In a study on an Alzheimer’s disease model using SAMP8 (accelerated senescence of mouse proprio 8) mice, resveratrol supplementation presented helpful effects by starting the AMPK and Sirt1 pathway, inducing increased cell survival and therefore effect on longevity [112]. Additionally, a recent study proved that the antioxidant abilities of this phytochemical could decrease H_2_O_2_-induced senescence in bone marrow mesenchymal stem cells, in part through modulation of the avian viral oncogene homolog A-v-rel-reticuloendotheliosis and Sirt1 [113]. Therefore, the anti-inflammatory and antioxidant properties of resveratrol give this compound an anti-aging capability, which positively affects TeLe and slows down telomere shortening, resulting in the possible prevention of age-related diseases [114]. One study proved that low doses of resveratrol and gallic acid increased the expression of the hTERT gene in the HepG2 hepatocellular carcinoma cell line, probably through induction of the SIRT1/Nrf2 signaling pathway [115].

Green tea (*Camellia sinensis*) is certainly the most widespread flavored and functional drink in the world. It is rich in polyphenols (especially catechins), tocopherols, carotenoids, minerals, ascorbic acid, and other phytochemicals. The catechin derivatives of green tea include, epigallocatechin, epicatechin, epicatechin gallate, and epigallocatechin gallate (EGCG), which has the greatest anti-inflammatory and anticancer potential. Notably, catechins in green tea have been explored for their ability to prevent a variety of cancers [116]. These compounds act in various ways, including quenching harmful reactive nitrogen and oxygen species, acting as metal chelators, and inhibiting the enzymes lipoxygenase, cyclooxygenase, and xanthine oxidase [116,117]. A cross-sectional study involving Chinese men and women associated TeLe in elderly men with frequent and regular use of green tea [118]. Another study showed that regular tea drinkers have longer telomeres in their blood cells than those who drink less tea [119]. 

A healthy lifestyle with a diet rich in vegetables and fruits combined with physical exercise and a lower body mass index is in fact linked to longer telomeres [120].

A further study proved that EGCG and quercetin, with strong antioxidant effect, could prevent apoptosis of cardiac myocytes by retarding telomere shortening and the loss of telomere repeat-binding factor 2 expression [121]. 

A high nutritional intake of the antioxidants α, β-carotene, and α-tocopherol in beneficial Japanese adult’s diet defends buccal mucosal cells from telomere shortening [122]. Also, it was proposed that a high eating of carotenoid-rich foods could play a role in defending telomeres against oxidative alterations [123].

The consequence of a highly concentrated polyphenol extract of bergamot fruit (*Citrus bergamia*) on UVB-induced photoaging was evaluated by investigative the expression of inflammatory cytokines, alterations in telomere length/telomerase, and cell viability in human keratinocytes HaCaT immortalized. Photoaging is the addition of chronic extrinsic damage made by ultraviolet radiation to intrinsic aging and accounts for many age-associated variations in skin appearance. The results suggested that this polyphenol extract protects HaCaT keratinocytes from UVB-induced oxidative damage and photoaging markers in a dose-dependent way and could be a convenient supplement in skin care products. Together with its antioxidant ability, the highly polyphenol concentrated extract of the bergamot fruit seems to moderate the fundamental cellular signal transduction pathways that lead to antiproliferative, anti-aging, and immunomodulatory responses, also linked to TeLe [124] (Table 1).

Therefore, investigations suggest that polyphenols, with their anti-inflammatory and antioxidant capabilities, can influence TeLe and avert its shortening and therefore have powerful anti-aging capabilities [9]. Polyphenols can decrease the inflammatory response, modulate nutrient sensing pathways, and cause selective apoptosis of senescent cells. These biological events are important promoters of diseases development, as they become dysfunctional with advancing age [125]. Therefore, the precise elucidation of the molecular mechanisms of these phytochemicals in the modulation of biological phenomena related to aging becomes challenging due to the complexity of biological systems, where diverse biochemical aspects contribute to the establishment of this phenotype [114]. Currently, to increase the activity of the telomerase enzyme, slow down the shortening of telomeres, and increase the lifespan, the use of natural antioxidant supplements has been proposed [102]. It has been proposed that polyphenols, with their antioxidant and anti-inflammatory capabilities, can influence TeLe and effectively slow down its shortening. The effects of dietary antioxidants on telomere function have shown that diet is a significant factor in finding TeLe status. In this regard, some researchers have proved how the length of leukocyte telomeres can be significantly improved in subjects who follow a diet rich in olive oil [62,126].

## 6. Polyphenols Bioavailability and Interaction with Microbiota

The usual daily dietary intake of polyphenols is between 0.1 and 1.0 g. However, it is important to underline the poor bioavailability of polyphenols in humans due to the reduced absorption at the intestinal level and the rapid biotransformation that these molecules undergo which helps their urinary excretion. With some exceptions, polyphenolic aglycones can be moderately absorbed in the small intestine by passive diffusion much better than their glycated counterparts [127], although high quantities continue to be eliminated in the large intestine [128].

Bioavailability is generally defined as the fraction of an ingested nutrient or compound that reaches the systemic circulation and the specific sites where it can exert its biological activity. The health effects of polyphenols in humans depend on their absorption, distribution, metabolism, and elimination. Therefore, to prove the efficacy of these dietary phytochemicals in longevity, it is useful to better define their bioavailability [74,129,130]. Their speed, the extent of absorption, and the type of their metabolites present in the various tissues certainly depends on the chemical structure. Polyphenols can be present in plants with the chemical structure of glycosides, aglycones, polymers, or esters. As already mentioned, intestinal enzymes and the microflora present in the colon can hydrolyze polymers, esters and glycosides, and the products of this enzymatic digestion can be absorbed; however, aglycones can be absorbed from the small intestine. The molecular weight, stereochemistry, lipophilicity, the presence of groups capable of hydrogen bonds are all structural characteristics that can influence their transport and permeability [131]. Through the gastrointestinal tract, low-molecular-weight derivatives are rapidly absorbed [132]. However, it must be underlined that many phenolic molecules can be absorbed at a rate of 0.2–44%, and the concentration of metabolites circulating in the plasma can be very low. In conclusion, it is possible to state that there are numerous factors that can interfere and influence the bioavailability of polyphenols assimilated through plant foods [133,134].

Catechins are unstable phytochemicals under physiological conditions and can generally be rapidly degraded or metabolized through interactions with the hydroxyl groups present on the phenolic rings typical of their chemical structure. After drinking a cup of tea, only a small part of the catechins is absorbed by the intestinal microvilli. In one study, it was shown that only 1.68% of ingested catechins were present in human plasma, 1.1% in urine and 0.42% in feces [133]. Phase II enzymes, catechol-*O*-methyltransferase, UDP-glucuronosyltransferase, and sulfotransferase are involved in the degradation of ingested catechins which occurs mainly in the small intestine and liver [135]; however, other catechins enter the colon. Therefore, the main metabolites present in humans are represented by glucuronide and sulphate conjugates, methylated catechin conjugates, phenolic acid catabolites and ring fission products due to the action of the microbiota [136,137].

Quercetin is absorbed in minimal quantities at the gastric level as the small intestine is the main site of its absorption. The absorbed unit is the aglycone which requires the removal of the glycosidic groups before absorption. It is important to underline how quercetin glycosides are generally more bioavailable than the aglycone which is more insoluble in the lumen of the intestine. Because brush border enzymes are specific for glucose, quercetin glycosides are absorbed more rapidly than other types of glycosides. After absorption, quercetin is methylated, glucuronidated, and/or sulfated. Quercetin derivatives that are not absorbed in the small intestine, such as rutin, pass into the colon and there undergo deglycosylation by enzymes secreted by the microbiota. Generally, bioavailability presents interindividual variations; genetic polymorphisms, dietary history, and variations in microbiota metabolism are believed to have a determining role in digestion and absorption [138]. Among flavonoids, the fate of anthocyanins ingested through food follows a unique pattern different from that of others. Such polyphenols could be absorbed by the stomach and intestines. Some anthocyanins are absorbed more easily when taken with rich, very sweet fruit. Anthocyanins appear rapidly in the plasma, brain, and bile. Metabolites and their actions are not well understood. Monoglucuronides and sulfates predominate in the urine. Some anthocyanins can reach the large intestine in significant quantities and undergo digestion by the action of the microbiota. In turn, these digestion-derived metabolites may contribute to the beneficial properties related to anthocyanins [139].

Since polyphenols are widely changed and the forms that appear in the blood and tissues are predominantly very different from those present in plants and therefore consumed through plant foods, scholars are also trying to explore the potential biological activity of polyphenol metabolites, also investigating the interaction with the microbiota.

The human gut microbiome includes 10^14^ microorganisms including protozoa, bacteria, viruses, fungi, and viruses, which are commensal with the intestinal tract [140,141]. Bacteria are the most studied group and overall, the predominant bacterial groups are the Gram-negative Bacteroidetes and Gram-positive Firmicutes [142]. In recent years, many scientific papers have proved how the microbiota can be distinguished into different enterotypes, each enriched by bacterial genera, but which nevertheless all seem to share a high functional uniformity. This uniformity is present regardless of different host properties, such as sex, age, nationality, and body mass index [143]. Many of the microorganisms forming the intestinal microbiota live in the most distal parts of the digestive tract, where their biomass reaches 10^11^ cells per gram of content. Microbes in the distal gut contribute to host health through the synthesis of many essential vitamins and amino acids, as well as the synthesis of metabolites from undigested food molecules in the small intestine [141].

In recent years, a possible correlation between aging and the host’s intestinal microbiota has become a widespread topic in biomedical research. The gut microbiota can act as a barrier against pathogens, stimulate the host to synthesize antibacterials, and help maintain host health [140]. The host’s diet is one of the critical factors in regulating the composition of the microbiota and different dietary habits influence its composition [144]. Recent studies have shown that both polyphenols supplied to intestinal bacteria through foods, and the aromatic metabolites produced, can modulate and cause fluctuations in the composition of the microbiota through selective prebiotic effects and antimicrobial activities. The synthesis of bioactive metabolites derived from polyphenols and the modulation of the colon microbiota can have positive effects on the health of the host, the mechanisms of which, however, are still being studied [145]. Polyphenols taken from plant foods and the metabolites derived from them modulate the intestinal microbial balance through the stimulation of the growth of beneficial bacteria and the inhibition of pathogenic bacteria, exerting effects like prebiotics. However, data on the impact of polyphenols on the intestinal microbiota and their mechanisms of action in humans are still being studied. Furthermore, a better understanding of the relationship between dietary phenolics and the gut microbiota by combining metagenomic and metabolomic studies could supply further insights into the effects of phytochemicals on health [145]. However, the intestinal microbiota, the host and polyphenols interact with each other. The intestinal microbiota degrades polyphenols resulting in the synthesis of metabolites which have been recognized as having an antioxidant and/or anti-inflammatory action; short-chain fatty acids and other molecules are produced which can support the integrity of the intestinal barrier, inhibiting intestinal inflammation and stimulating the production of neurotransmitters involved in the control of some activities of the central nervous system.

Therefore, the mechanism of polyphenols, including their ability to slow down the shortening of telomeres, is related to microbiota actions [146]. Once in the colon, some polyphenols undertake microbial fermentation producing small polyphenol bioactive metabolites, which, in part, might handle their health benefits [147]. Polyphenols’ metabolic fate is thus affected by different factors, such as interindividual variations in polyphenol metabolism caused by genetic polymorphisms of transporters, metabolic enzymes, efflux pumps, and the bidirectional communication with the gut microbiota appears to be the primary driver of this interindividual difference [148,149,150].

## 7. Conclusions

The close relationship between nutrition and health is now a fact. It is known that the negative effects of an inadequate diet can lead to the risk of developing chronic diseases. Eating habits can change people’s psychophysical state, improving it by at least 300 or 400%, while genetics accounts for 30–40%, so what we eat is decisive. Telomere length is a biomarker of organic aging. Due to partial duplication of linear chromosomes by DNA polymerase, telomeric repeats at the ends are lost with each cell division. The development of some age-related diseases is linked to telomere dysfunction. Inflammatory processes that cause an increase in cell turnover, increasing the frequency of cell divisions, contribute to a faster attrition of telomeric repeats. Damage to telomeric DNA due to oxidative stress or reduced obtainability of nucleotide precursors causes a sudden shortening of the telomeres. Antioxidant and anti-inflammatory nutrients can slow down the erosion of telomeres. Polyphenols are still, and will certainly continue to be, probable potential candidates in the pharmaceutical and medical fields for promoting human health, preventing, and treating several syndromes. If we consider that only 15 percent of the approximately 300,000 species of terrestrial plants described have been systematically studied for their biological capabilities and/or phytochemical profiles, from time to time, with ancient methods or in a non-systematic and exhaustive way, a vast field of research still appears to be open for exploration on health-promoting polyphenols.

The ability of many phytochemicals to slow down telomere length shortening, as proposed in this review, is founded on present knowledge and will need to be further investigated in future research.

## Figures and Tables

**Table 1:** The reviewed main studies about bioactive phytochemicals and telomeres.

**Plant Food**	**Bioactive Phytochemicals**	**Outcomes**	**Ref.**
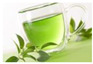	Epigallocatechin-3-gallate (EGCG)	Longer telomeres	[116,119]
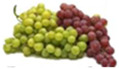	Resveratrol (trans-3,5,4′-trihydroxystilbene)	Increase in the telomerase enzyme activity	[105,109]
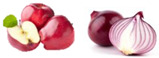	Quercetin (3,3′,4′,5,7-pentahydroxyflavone) + Epicallocatechin-3-gallate (EGCG)	Slows down telomere shortening	[121]
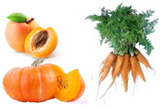	α, β, carotene α-tocopherol	Slows down telomere shortening	[122,123]
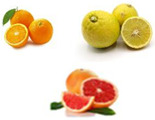	Naringin Hespertin	Slows down telomere shortening	[124]
**Diet**	**Bioactive Phytochemicals**	**Outcomes**	**Ref.**
Mediterranean diet (rich in polyphenolic food)	Phytochemical mixture (mainly polyphenols)	Slows down telomere shortening and/or increases the telomerase enzyme activity	[11,22,28,32,34,53,54,62,64,65,66,67,68,70]
Plant-based diet	Phytochemical mixture	Slows down telomere shortening and/or increases the telomerase enzyme activity	[15,26,27,33,35,37,38,39,40]
